# In humans, only attractive females fulfil their sexually imprinted preferences for eye colour

**DOI:** 10.1038/s41598-020-62781-7

**Published:** 2020-04-07

**Authors:** Paola Bressan

**Affiliations:** Dipartimento di Psicologia Generale, University of Padova, 35131 Padova, Italy

**Keywords:** Sexual selection, Human behaviour

## Abstract

Early exposure to parental features shapes later sexual preferences in fish, birds, and mammals. Here I report that human males’ preferences for a conspicuous trait, colourful eyes, are affected by the eye colour of mothers. Female faces with light (blue or green) eyes were liked better by men whose mother had light eyes; the effect broke down in those who had felt rejected by her as children. These results, garnered on over one thousand men, complete those of a symmetrical study on one thousand women, painting a fuller picture of human sexual imprinting. Both men and women appear to have imprinted on their opposite-sex parents unless these were perceived as cold and unjustly punitive. Birds require strong attachment to sexually imprint—a constraint in place to reduce the perils of acquiring the wrong sort of information. Parents who form no bond with their offspring may fail to be recognised as appropriate parental imprinting objects. Consistent with human females being, as in most of the animal kingdom, the choosier sex, imprinted preferences were displayed by both sexes but translated into real-life partner choices solely in women—*attractive* women. Apparently, not all of us can afford to follow our own inclinations.

## Introduction

There is a road from the eye to the heart that does not go through the intellect.

–G. K. Chesterton, The Defendant, 1901^1^

## Templates for choosing a partner

The preference for certain mates over others can be learned early in life^2^. Across a wide range of species, young animals form a template of what their future sexual partners ought to look like by “imprinting on” their father, their mother, or a random individual of their parents’ generation; or more precisely, on some trait carried by them. Such a trait turns into a signal that, at some future time, will indicate which potential mates are better than others. For example, females imprint on the odour and colour of their father in sticklebacks^3^; on those of their mother in cichlids^4^; and on the foreleg colour of the first adult male that, prematurely, courts them in wolf spiders^5^. Thus, all three imprinting modes are observed in nature. Why a particular one gets an edge over the others is an interesting point, that like many other interesting yet complex or counterintuitive points can be understood with the help of mathematical models^6^.

For the excellent reason that females are normally the choosy sex, all such models^7–9^ are built on the assumption that only females imprint. As they provide much greater parental investment than males do, females have evolved to be particularly selective when it comes to picking potential mates^10,11^. Rather than just waiting about, members of the nonchoosy sex will court the choosers and compete among themselves to be chosen. Models show that, on these grounds, the best strategy for females is that of imprinting on their fathers (^7,8^; but see^9^). Fathers have proven to be capable of surviving to reproductive age *and* be sexually successful—that is, they have won the competition with other males. By imprinting on a paternal feature, thus, daughters are likelier to end up picking a mate whose offspring will survive and be sexually successfully, too.

This argument contrasts fathers with both mothers and random individuals. The latter have only the distinction of having survived to adulthood to show for themselves. Mothers have also successfully reproduced, true, but unlike fathers they have not triumphed in a peer competition (remember we are talking about species with exclusive female choice). Females who use mothers or others as models for a partner, thus, are less likely to end up with a successful mate than are females who use fathers. Naturally, fathers are not always around, which goes a long way to explain why some species do imprint on mothers. Maternal imprinting might also be favoured in monogamous mating systems with a great deal of extrapair paternity—one reason being that daughters would often be imprinting on a social father who is not their biological father, and possibly has not reproduced at all^9^. As to female spiders, they may have no other option than imprinting on a chivalrous mature male (not right after hatching but as “teenagers”^5^) since they receive no care whatsoever from parents; the same goes for fruit flies^12^.

When males are themselves the choosy sex, as in numerous species of fish, the imprint-on-the-winner logic can be transferred to them: sons ought to imprint on mothers (see^13^). This time around, it is mothers who have emerged from the struggle with other females to be chosen by a male. Yet the notion that sexes are either entirely choosy or entirely competitive has been challenged even in species with uniparental care (reviewed in^14^), and surely falls short of describing our own mating system. In humans, females are arguably the choosy sex (e.g.^15^) but also engage in considerable courting and competing, and males court and compete a great deal but do some rejecting too (e.g.^16^).

## Do humans sexually imprint?

In fact, some studies suggest that humans of both sexes tend to be paired with people who resemble their opposite-sex parent. Unfortunately, most such evidence is either open to alternative explanations or counterbalanced by failures to replicate. For example, about 60% of children of ethnically different parents marry, and re-marry, into their opposite-sex parent’s ethnic group (according to the marriage records of 980 twice-married individuals in Hawaii^17^). However, this association could arise from having inherited the preferences of one’s same-sex parent^18^ just as well as from sexual imprinting on one’s opposite-sex parent. By marrying a black man, the daughter of a black father and a white mother may be repeating her mother’s choice because she has inherited her mother’s preferences for black men, not because she has imprinted on her black father. This objection also applies to the findings that women whose fathers have a hairy chest tend to have partners with a hairy chest, too^19^, and that the eye colours of partners and opposite-sex parents are more similar than expected by chance^20^. Two studies^21,22^ reported above-chance facial resemblances between people’s partner and opposite-sex parent, especially for individuals who had enjoyed a good relationship with that parent as children. Yet both studies asked neutral judges to rate the resemblance of four possible spouses of a person to that person’s parent, so the particular set of three nonspouses that happened to accompany the actual spouse would weigh heavily on the outcome. These results, in fact, have not been replicated^23,24^.

Of course, human couples are the product of environmental contingencies and of preferences other than those for certain visual features. Such matters add noise; and noise can dilute true effects but also, in small samples especially, create spurious ones. So, given that imprinting sways mate choices by shaping mate *preferences*, studying mate preferences directly rather than, or along with, actual mate choices seems best. From this angle, however, the evidence is not much clearer. For example, no preference for hairy chests was found in women whose fathers had a hairy chest^25^, casting doubt on whether the similarity in hairiness between partners and fathers^19^ is truly due to sexual imprinting. Opposite-sex parent’s height and body type faintly affect, respectively, preferences for height^26^ and body type^27^ in an ideal partner, but do not count in actual partner choices^27^.

As to preferences for faces that resemble one’s parents in the context of mate choice, the literature only offers three studies. In a sample of 49 women, those whose self-assessed childhood relationship with their father was above the median preferred men’s faces that resembled him—in the proportion of certain features, such as nose height relative to face width, but not others, such as nose width relative to face width^28^. Assuming that this finding (obtained on 23 women) is not a false positive, simpler explanations than sexual imprinting are available, because neither self-resemblance nor mother-resemblance were either tested or controlled for. Thus, the women may have just preferred self-resemblant faces, with an effect of paternal relationship surfacing because fathers have a better rapport with children who resemble them more^29,30^—which would produce an *apparent* preference for good fathers’ faces. This account would predict not only that father- (i.e., self-) resemblant male faces are liked better than other male faces, but that father- (i.e., self-) resemblant *female* faces are liked even better relative to other female faces^31^; a test that cannot be done on these data, because participants only saw male faces. Alternatively, the effect could stem from transference—the automatic inclination to evaluate a stranger who resembles an important person in one’s life as as likeable or unlikeable as that person^32,33^. Men who resemble one’s father would be liked better than men who do not, but only if the daughter had had a good relationship with her father in the first place. Unlike sexual imprinting, transference may predict that male faces who resemble one’s likeable mother would be liked better too—again, a check that one cannot perform here, because resemblance to mothers was not measured.

The remaining two studies both relied on facial images manipulated to resemble the participant or parent(s) but reached very different conclusions. In the first^34^, 24 men from an Indonesian village appeared to like a female face resembling their own mother *less* (rather than more) than other men did. In the second^35^, 96 participants failed to show a preference for faces of potential partners that had been manipulated to resemble either the participants themselves, their mother, or their father, relative to faces manipulated to resemble a stranger. Despite not being above chance, however, the preference for faces that resembled the opposite-sex parent was larger in participants who had experienced less rejection (but not less overprotection or more emotional warmth) from that parent during childhood.

Altogether, then, the evidence in favour of human sexual imprinting is inconclusive. One reason is likely to be the reliance on small samples—an obligate choice in studies where photographs of each participant and parent require to be measured or manipulated. Internet surveys permit larger samples, but can produce confounded data if information that may turn out to be relevant is not collected. As a case in point, one such survey run on an English-speaking sample of 394 men and 303 women of undetermined ethnic backgrounds^20^ found correlations in eye colour between participants’ partners and opposite-sex parents (and between participants and their partners, too). Yet eye colour is also a proxy for ethnicity and ancestry—both major drivers^36,37^ of positive assortative mating, that is, the tendency to choose mates similar to oneself. Thus, the mere inclination to marry into the ethnic group of one’s opposite-sex parent^17^—whether due to sexual imprinting or inherited preferences or cultural customs—would be enough to create such correlations even if nobody imprinted on eye colour or even cared about it. For example, if the daughters of black fathers and white mothers tended ever so slightly to marry black men, both father-partner and self-partner (i.e., assortative-mating) similarities in eye colour would arise as indirect and meaningless consequences.

Still, eye colour is a feature that, being exceptionally salient and—unlike facial hair—not appreciably sex-linked^38^, would be ideal for both males and females to imprint on. We^39^ recently investigated eye colour preferences *and* choices on a sample of over one thousand women of the *same* ethnicity. Daughters of fathers with light (blue or green) eyes showed a stronger preference for light-eyed men than daughters of fathers with dark (brown or dark brown) eyes did. The effect was there in the absence of assortative mating for eye colour, hence it was not a byproduct of it; it was specific to fathers, mothers’ eye colour being irrelevant, hence it did not arise from mere familiarity; it was modulated by the quality of the early relationship between father and daughter, hence it was very unlikely to simply reflect inherited maternal preferences; it extended to both long- and short-term potential partners and to actual, real-life partners too, hence it was robust and ecologically relevant. After examining the tenability of alternative explanations, we concluded that these findings provide compelling evidence for paternal imprinting in human females.

Here I report a perfectly symmetrical study, carried out with similar materials and the same procedure on a sample of over one thousand men. This study’s results, combined with those garnered on women and with a reassessment of earlier evidence, paint a fuller picture of sexual imprinting in humans.

## Methods

### Participants

The study was conducted online. Participants were recruited mostly via advertisments posted on Italian universities’ online social networks and other social media. Most of the links redirected men who reported being homosexual, or bisexual but with a preference for male partners, to a separate study. Participants who failed to disclose their sexual orientation were excluded from the dataset. The final sample was composed of 1440 men (median age = 23 years, range = 18–73 years). Of these, 734 reported having a partner. The experimental protocol was approved by the Psychological Research Ethics Committee of the University of Padova and data were collected in accordance with the relevant guidelines and regulations. Informed consent was obtained from all participants.

### Materials and procedure

**Figure 1:**
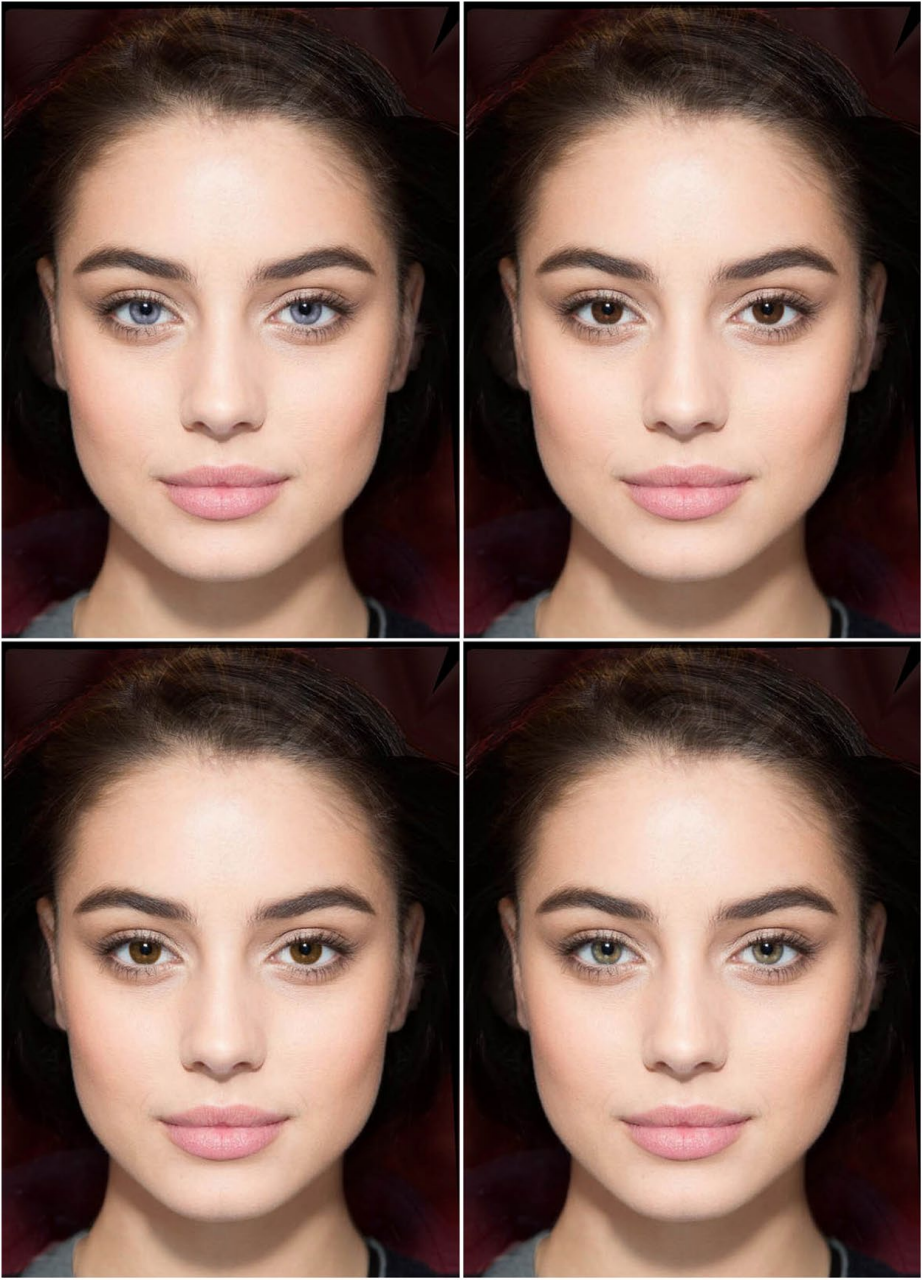
Example stimuli. Participants saw pairs of identical female faces, one with light and the other with dark eyes. For each face, eye colour combinations were either blue/dark brown (top) or brown/green (bottom); each participant saw only one of the two. The face portrayed here has been created digitally for purposes of illustration^65^; the study presented photographs of real women. Image copyright by Paola Bressan.

The method was identical to that used by Bressan and Damian^39^ in their study on women, except that the stimuli were 10 colour photographs of attractive young women’s, rather than men’s, faces. Photographs were digitally modified in PortraitPro, creating four versions of each face which differed only in eye colour. As in^39^, these were always shown in pairs (Fig. 1): blue/dark brown (with blue on the left) and brown/green (with green on the right). Each face was presented in the blue/dark brown combination to half of the participants and in the brown/green combination to the other half; everybody saw 5 blue/dark brown and 5 brown/green pairs. The full set of 10 pairs was shown once in a long-term relationship context and once in a short-term one, with context order counterbalanced across participants. Participants were asked, “If you were looking for a long- (short-) term relationship, which of these two people would you prefer?”. Possible responses were “the one on the left” and “the one on the right”.

In a questionnaire presented at the end, participants were asked about their own eye colour and that of their parents and current partner, if they had one. Possible choices were “dark brown”, “brown”, “hazelnut (very light brown)”, “green”, “grey”, blue”, and “other” (with the option to specify). Participants also reported how much they had felt rejected by each parent during childhood via three representative items of the short form of the EMBU Rejection Scale^40^: “I was treated as the ‘black sheep’ or ‘scapegoat’ of the family”, “She [he] would punish me hard even for small things”, and “It happened that she [he] was cold or angry with me without letting me know the reason”. Each behaviour’s frequency was estimated on a 0–3 scale from “no, never” to “yes, very often”.

### Data analysis

All data coding and selection choices were identical to those made by Bressan and Damian^39^. For correlational analyses, participants’ eye colours were coded from light to dark: 1 = blue, 2 = green, 3 = very light brown, 4 = brown, 5 = dark brown. For analyses on preferences for virtual partners with *light* (blue or green) vs *dark* (brown or dark brown) eyes, participants’ blue and green eyes were coded as “light” whilst brown and dark brown eyes were coded as “dark”.

Analyses involving parents and children excluded participants who reported having cohabited with the relevant parent for 1 year or less (on the grounds that they would have had little or no opportunity to imprint on the parent) and participants who did not provide cohabitation information. This led to exclusion of 28 men for analyses involving mothers and 21 for those involving fathers. However, the pattern of results was the same with and without these exclusions. Of the 1440 non-homosexual men that completed the study, 46 (3%) reported being bisexual and 25 (1.7%) were either not Italian or raised but not born in Italy. The pattern of results remained the same if the data of the former or latter participants were discarded from the analyses. Only results directly relevant to the topic of sexual imprinting are reported; the complete result set of the analyses described in this article can be found in the Supplementary Material (see Data Availability section).

## Results

### Sexual imprinting affects the mate *preferences* of men and women in similar ways

Men’s preference for light-eyed women was computed as the number of choices of light-eyed faces relative to the total number of choices. This index could range from 0 (light-eyed face is never chosen) to 1 (light-eyed face is chosen every time).

A repeated-measures ANOVA was carried out on these preferences, with a within-subject factor of relationship context (long-term, short-term) and between-subject factors of own and maternal eye colour (light, dark). The preference for light eyes was stronger in men who had light eyes themselves, *F*(1, 1083) = 22.027, *p* < 0.0001; being unrelated to the issue of sexual imprinting, this large effect will be explored in the future and is not further discussed here. (Note, incidentally, that this obvious preference of light-eyed men for light-eyed women failed to be converted into actual partner choices. There was no significant correlation between the eye colours of the men and of their current partners, whether colour was measured on the 5-point darkness scale from blue to dark brown, Spearman’s *r* = −0.017, *p* = 0.661, *N* = 651, or coded as light vs dark, *r* = −0.048, *p* = 0.272, *N* = 527. Thus, no positive assortative mating for eye colour was found in this sample.)

More to the current point, the preference for light eyes was stronger in men whose mother had light eyes, *F*(1, 1083) = 4.211, *p* = 0.040, suggesting that men sexually imprint on their mothers. There was no evidence that this effect depended on whether women were regarded as partners for a one-night stand or a lasting relationship (maternal eye colour × context, *F* = 2.084, *p* = 0.149). Replacing maternal with paternal eye colour showed that the light eyes of fathers played no significant role in affecting men’s preferences—either on their own or in any interactions, all *F*s < 1. These results mirror those found in women: light-eyed male faces looked more attractive to women whose father had light eyes, in both a short- and a long-term context^39^. This symmetry between the sexes also turned up in two separate multiple regressions on men and women’s overall preference for light eyes—with own, maternal, and paternal eye colour (light, dark) as independent variables. In both sexes, the opposite-sex parent’s eye colour significantly predicted the preference for light eyes (men: *p* = 0.011, women: *p* = 0.005) whereas the same-sex parent’s eye color did not (men: *p* = 0.605, women: *p* = 0.688).

In women, the impact of paternal light eyes depended on how much daughters had felt rejected by their father during childhood, with no significant effect found in participants in the highest tertile of paternal rejection^39^. Here the very same analysis was run on men; this was identical to the repeated-measures ANOVA described above except that it was carried out only on the men in the bottom and top tertiles of maternal rejection, so as to include an additional between-subjects factor of maternal rejection (low, high).

**Figure 2:**
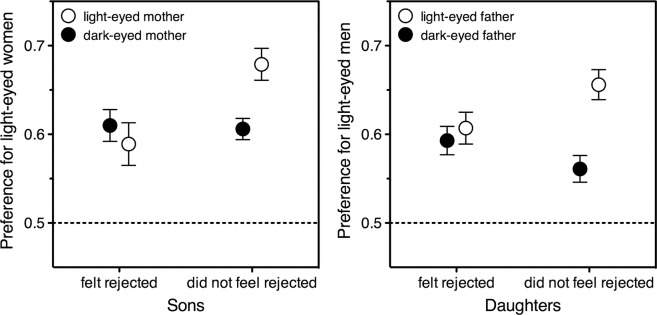
Mean preference for light-eyed partners as expressed by men (left panel) and women (right panel) in the top and bottom tertiles of rejection by the opposite-sex parent. Preferences are plotted as a function of opposite-sex parent’s eye lightness. Values higher than 0.5 (chance level, dotted line) represent a preference for light-eyed over dark-eyed potential partners; error bars indicate one standard error of the mean. Having a parent whose eyes are light (light symbols) or dark (dark symbols) makes no difference for participants who felt rejected (on the left of each graph, light and dark symbols are close together). However, having a parent with light eyes increases preference for light-eyed partners in participants who felt accepted (on the right of each graph, the light symbol is higher up than the dark symbol). Left panel: Men’s preferences as a function of mother’s eye lightness and rejecting attitude (*N* = 722). Data source: current study. Right panel: Women’s preferences as a function of father’s eye lightness and rejecting attitude (*N* = 627). Data source: Bressan & Damian^39^.

Own eye colour did not interact with maternal rejection, *F* < 1, but mother’s eye colour did, *F*(1, 624) = 5.176, *p* = 0.023. Figure 2, left panel, depicts this interaction. As shown by separate ANOVAs, the light eyes of rejecting mothers did not significantly increase the sons’ preference for light-eyed partners, *F* < 1, whilst the light eyes of nonrejecting mothers did, *F*(1, 467) = 11.555, *p* = 0.001. This mimics the effect found in women (Fig. 2, right panel). Note how alike the two charts are: in both sexes, parental light eyes increase the preference for light eyes solely in people who did not feel rejected by the parent. Here, for clarity, parental eye colour and rejection are both treated as dichotomous variables (parents with light vs dark eyes; children who felt least vs most rejected). However, the interaction remains significant if full ordinal information is retained (eye darkness: 1–5; rejection: 0–9). A multiple regression analysis^41^ on the overall preference for light eyes yields similar results for men and women (opposite-sex-parent’s eye darkness × rejection, men: beta = 0.23, p = 0.004; women: beta = 0.21, p = 0.004). In both men and women, twin multiple regressions show that the opposite-sex-parent’s eye darkness separately predicts long-term and short-term preferences (see Supplementary Material).

To compare the “imprintabilities”^42^ of the two sexes, finally, I analysed the data for men (source: current study) and women (source: Bressan & Damian^39^) together. All individuals with light or dark eyes whose opposite-sex parent had light or dark eyes and who had cohabited with such parent longer than 1 year (1087 men, 873 women) were included in a repeated-measures ANOVA. The within-subject factor was relationship context, and the between-subject factors were sex, own eye colour, and opposite-sex-parent eye colour. Crucially, the effect of opposite-sex-parent eye colour, *F*(1, 1952) = 14.073, *p* = 0.0002, did not interact with sex, *F* < 1. That is, there was no evidence that the overall strength of sexual imprinting on light eyes was different for men and women.

### Sexual imprinting affects the actual mate *choices* of men and women in different ways

Imprinting on maternal eyes swayed men’s preferences but not their choice of a partner in real life. Participants’ partners and mothers did not have the same eye colour more often than expected by chance. This was true both across two eye-colour categories (light, dark: *X*^2^(1) = 0.010, *p* = 0.919) and all four of them (blue, green, brown, dark brown: *X*^2^(9) = 12.301, *p* = 0.197, *N* = 526). This came as something of a surprise, given that, in women, fathers and partners did feature the same eye colour significantly more often than expected^39^.

Thus, an opposite-sex parent with light eyes increased men’s and women’s preferences for light eyes in similar ways, but these preferences showed up only in women’s, and not men’s, partner choices in real life. One explanation for this puzzling inequity could be that it is the females (the choosier sex, let’s not forget), and not the males, who have the final say over the pairings. To test this idea, I reasoned that the choosiest of the choosy might succeed in realising their own preferences best. Both theory and data indicate that it is the most attractive individuals who can afford to be choosiest^16,43^. The questionnaire that had been presented to both women and men, alongside questions bearing on the specific hypothesis of parental imprinting, included others devised for a separate study on individual differences. One such question asked participants to evaluate their own physical attractiveness on a 0–10 scale. I added this variable (split along the median, to obtains subsamples as large as possible) as a between-subjects factor, together with own and paternal eye colour, to the repeated-measures ANOVA on women’s preferences for light eyes. Paternal eye colour, *F*(1, 848) = 8.747, *p* = 0.003, did not interact with participant’s attractiveness, *F* < 1. That is, the impact of paternal imprinting on preferences was not significantly different in women whose self-assessed attractiveness was above the median and below the median.

**Figure 3:**
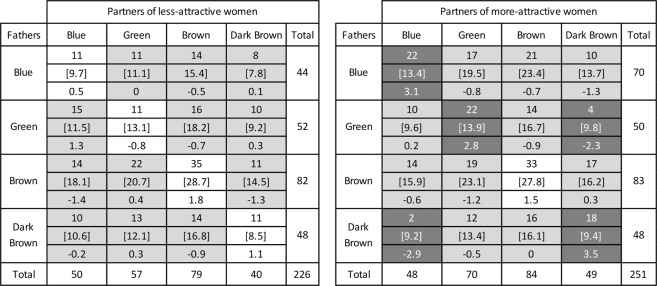
Distribution of father-partner eye colour pairs in women below (left panel) and above (right panel) the median in self-assessed physical attractiveness. From top to bottom for each eye colour category: observed frequencies, expected frequencies (in brackets), and adjusted standardised residuals. Standardised residuals above 2 (below −2), shown in white on a dark background, indicate that the observed frequency is significantly greater (smaller) than the expected frequency. In each panel, cells along the diagonal from the upper left to the lower right refer to the number of participants whose father and partner had the same eye colour; black text on a white background indicates no difference from the number expected by chance. Data refer to partnered women who had cohabited with their father longer than 1 year, whose father’s and partner’s eye colours were either blue, green, brown, or dark brown, and who provided a self-attractiveness rating (*N* = 477). Data source: Bressan & Damian^39^.

Next, I computed the distribution of observed and expected father-partner eye colour pairs in the same two subsamples. In women whose self-assessed attractiveness was *below* the median (Fig. 3, left panel), partners and fathers never had the same eye colour more often than expected by chance, *χ*^2^(9) = 6.609, *p* = 0.678, *N* = 226. In women whose self-assessed attractiveness was *above* the median (Fig. 3, right panel), however, they did, *χ*^2^(9) = 31.314, *p* = 0.0003, *N* = 251. In particular, the father-partner eye colour combinations blue/blue, green/green, and dark brown/dark brown all occurred significantly more than expected, whereas the combinations green/dark brown and dark brown/blue occurred significantly less than expected. Yet there was no trace of this effect in men’s data: *none* of the mother-partner same-colour combinations occurred significantly more often than expected, not even in the subsample of men whose self-assessed attractiveness was above the median (see Supplementary Material).

## Discussion

The results of this large study on men, together with those of the twin study on women^39^, show that people who prefer an opposite-sex partner have sexually imprinted on their opposite-sex parent. From these data, human sexual imprinting emerges as equipped with three notable features—none of which, as far as I am aware, has been directly explored in other species. First, both females and males avoid imprinting on parents who appear cold and unjustly punitive. Second, both females and males use the same imprinting model when selecting social partners and extrapair mates (as opposed to being driven by imprinted preferences in one or the other choice only: see^9^ for mathematical modelling of all three scenarios). Third, actual mate choices are aligned with imprinted preferences only in attractive females.

### Humans do not sexually imprint on parents they have not bonded with

Young zebra finches raised by Bengalese finches proceed to strongly imprint on them (going on to seek Bengalese finches as mates) even when surrounded by adults, and sharing the nest with siblings, of their own species^44^. Immelmann interpreted this observation as evidence that the determination of sexual preferences requires an “emotional attachment of exceptional strength”^44^^, p. 7^, as that which is established through parental care. Individuals with whom no such bond is formed fail to serve as imprinting objects even when they provide, as siblings do, “a certain amount of personal attachment, too”^44^^, p. 6^.

In natural conditions, the young’s “emotional attachment of exceptional strength” will be addressed to the caregiver and the caregiver will be the parent. Therefore, this constraint ensures that the individual who is enlisted as a template for future mates is indeed the parent. Clearly this minimises the risk of acquiring the wrong sort of information; as it would occur, for example, if the young were able to sexually imprint on an unrelated animal that just happened to hang around a lot, without ever interacting with the young themselves. Yet whilst parental care and “emotional attachment of exceptional strength” can scarcely be separated in birds, they can and sometimes will be in humans. The data presented here suggest that this disconnection abolishes the impact of parental features on imprinted preferences. This impact does prove robust to some variation in parental emotional competence as experienced by the child—parents do not need to be perfect; nonetheless, perception of the parent as often cold, unfair, or unjustly punitive appears to lead to a breakdown of sexual imprinting. Drawing on Immelmann’s intuitions^44^, then, I propose that, at some instinctive level, parents who fail to provide strong emotional attachment are not recognised as such.

Note that this safeguard helps individuals pick mates which, like the original prototype, will not only successfully reproduce but be suitable parents to common offspring. A good parent, guaranteed as such by mandatory direct experience, supplies an internalised model of an opposite-sex individual who is proficient at family relationships. The flexibility injected into imprinted preferences by personal experience (“to imprint or not to imprint: that is the question”) is one which genetically transferred ones incline to lack (see also^45^).

Children’s impression of their parents as accepting or rejecting needs not necessarily change during development. So, that the perception of parents’ emotional ineptitude (their appearing cold and angry without reason, doling out punishment unfairly, treating the child as a scapegoat) can exert a veto on human sexual imprinting says little about whether the latter becomes completed by early infancy or gradually ossifies during childhood. Note however that, across both distant and tightly related species, the length of the period that permits sexual imprinting correlates closely with the duration of parental care^2^.

### Humans use the same imprinted template for lifelong and extrapair mates, and birds should too

Once they have become established, imprinted mate preferences extend to all potential sexual mates, in whichever context they are encountered. Expecting otherwise might in fact be naive. In our species, long- and short-term partners can be told apart only in retrospect (for example, brief relationships can be used to evaluate men as potential long-term companions or replacements for current ones^16,46^). One could even make a more general case that also applies to other socially monogamous creatures with biparental care, such as most species of birds, which practice sexual infidelity without deserting the social mate^47^. This logical argument holds for both humans and birds: if securing a mate with the physical traits of one’s parent furnishes some benefit to one’s offspring—as it must do to have evolved in the first place—imprinted preferences ought to cover both long- and short-term (including adulterous) partners, because offspring can be generated with either. This remains true whether such preferences serve to ensure fertile, sexy partners who will help produce sexy offspring, or partners genetically similar to oneself, who will help produce offspring that are well adapted to the local environment (“optimal outbreeding”^48,49^). I therefore expect birds, too, to display the same imprinted, parent-driven preferences in the choice of both life companions and extrapair mates. Some nonimprinted preferences could and should differ of course, consistent with the separate demands placed on life companions and extrapair mates. For example, the preference for resourcefulness in a mate will be more important in a lifelong partner than in an extrapair one, because it confers a larger benefit to offspring generated with the former than with the latter.

That imprinted preferences apply to all sexual mates squares with the discovery that people’s long- and short-term partners and ex-partners tend to have the same eye colour^50^. That this still shows up as a very feeble effect is rendered unsurprising by the findings discussed in the next section.

### Human (and avian) mate choices do not impartially reflect everyone’s imprinted preferences

On the assumption that mate preferences convert directly into mate choices, claims have been made of the existence, across several bird species, of an “imprintable” and a “nonimprintable” sex (reviewed in^42^). For example, males raised by foster parents of another species may end up being paired with the foster species much more often than do females, impelling researchers to conclude that males become imprinted and females do not. It has later been argued^51^ that such asymmetries may arise instead from a more decisive role of one sex in pair formation, and hence to the “acquiescent” sex’s preferences becoming obscured.

Our data provide two strong tests of this idea. First, imprinted preferences for light eyes were not significantly different in men and women. Yet women’s partners resembled fathers whilst men’s partners did not resemble mothers—meeting female but not male preferences. Of course, this may simply boil down to a difference in how the two sexes weigh the multiple criteria used in mate choice. For example, female zebra finches tend to be paired more often with mates with the “wrong” (nonimprinted) plumage than do males, but such mates turn out to be particularly good singers and wooers^42^. It is hard, however, to see why men should attach less weight to eye colour than women, especially considering that men place a greater value on the physical attributes of potential partners than women do^46^. A more sensible inference is that in humans—at least modern, western humans—males have a less dominant role in pair formation than females (cf^15^ for a similar conclusion); that is, males are the “acquiescent” sex just as they happen to be in most other animal species.

Second, imprinted preferences did not significantly differ between women whose self-assessed attractiveness was below the median and above the median. Yet the more attractive women turned out to be paired with partners who resembled fathers, in line with their own preferences, whilst the less attractive women’s preferences were apparently left unfulfilled. These findings fit well with the predictions of reasonably realistic models of mutual mate choice. In such models^16,52^, individuals set an “aspiration level” to decide whether or not a potential partner is good enough. The aspiration level reflects the individual’s self-assessed mate value. In some species mate value may simply parallel one’s age, and be straightforwardly “self-assessed” by the ticking of one’s biological clock^53^. In humans the process is likely to be more interesting, with an approximate mate value being figured out in the juvenile period—for example, by engaging in uncommitted flirting and recording expressions of interest and disinterest—and calibrated down the line^16,52^. The mating game is then played with “higher-quality” individuals steering clear of “lower-quality” ones, and “lower-quality” individuals adjusting their aspiration level to match their lower rank (see also^42,54–59^). The data presented in this article suggest that females’ self-assessed mate value changes their prospects of satisfying their sexually imprinted preferences for a certain trait, producing nonrandom mating relative to that trait. Attractive women were more likely to end up with men whose eyes had the same colour as the women’s fathers’.

The relationship between preference for facial health and partner’s facial health has been reported to be greater for more attractive women (attractiveness being estimated by independent observers^60^). That finding and the current ones dovetail neatly in sustaining the idea that women with higher mate value are better placed to translate their mate preferences into choices. Every bit as remarkably, in the same study neither an association between preference for facial health and partner’s facial health, nor an effect of own attractiveness, were found for the male partners of the same women. The authors interpreted this sex disparity in terms of physical attractiveness not being a good measure of a man’s mate value—as opposed, for example, to earning potential. This stands to reason. Yet physically more attractive men have higher success in both short-term^61^ and long-term^62,63^ mating and produce more children^62,63^; even their sperm is of better quality^64^. Thus they do have higher mate value, which makes it surprising that they seem unable to get a long-term partner with their favourite traits more often than do less handsome men. In the light of the new data presented here, I propose that the lack of an impact of men’s preferences for facial health on their choice of a partner is actually due to romantic couples receiving the final imprimatur from women, not from men.

This is not intended to imply that handsome males do not get females who are more attractive than average. Other things being equal they do, because attractive females (a) are bound to accept handsome males sooner than they accept plain males, and (b) are more interested in handsome males than other females are (shown in both humans^15^ and birds^58^). The implication is, instead, that a husband’s peculiarities—such as eye colour or facial health cues—will match the traits preferred by his attractive wife more often than a wife’s peculiarites will match those preferred by her attractive husband.

That not everyone pairs up with their first choice is painfully obvious in humans and would seem to be the case in other creatures, too. Female cockroaches, for example, become less choosy as they get older^53^, showing that they are cranking down their aspiration level based on a reality check. And of course, in none of the experimental studies conducted so far does *every* bird^51^, fish^3,4^, spider^5^, and fruit fly^12^ that is expected to have developed an imprinted preference get a mate with the favourite trait. Generalising our data from humans to other species, rather than the other way around as is usually done, one ought to conclude that the fairest members of the choosier sex can afford to act on their imprinted preferences; those a little less fair are forced to relax their standards and selectivity; and the members of the opposite sex can only show off, court, and hope. The nonchoosers’ pick of whom to compete for must still be guided by their own imprinted preferences, yet such preferences will largely be overshadowed by those of the choosers.

Sexual imprinting has been found wherever it has been looked for^51^—a testament to its being an extraordinarily popular, possibly quite ancient, mechanism for acquiring mate preferences. The findings discussed here suggest that humans are no exception. And also that, in our own species as in much of the rest of the animal kingdom, who ends up with whom is mostly determined by females.

## Data Availability

The data that support the findings of this study, along with all analysis scripts and outputs, are publicly available as Supplementary Material via the Open Science Framework and can be accessed at https://osf.io/ka8we.
